# A staged approach with initial tumor thrombectomy followed by hepatectomy for icteric-type hepatocellular carcinoma: A case report

**DOI:** 10.1016/j.ijscr.2019.09.030

**Published:** 2019-09-27

**Authors:** Takehisa Yazawa, Takehiro Ohta, Shun-ichi Ariizumi, Yutaka Takahashi, Ryota Higuchi, Masakazu Yamamoto

**Affiliations:** Department of Surgery, Institute of Gastroenterology, Tokyo Women’s Medical University, Tokyo, Japan

**Keywords:** TT, tumor thrombus, HCC, hepatocellular carcinoma, AFP, α-fetoprotein, PIVKA-II, vitamin K absence or antagonist-II, CA19-9, carbohydrate antigen 19-9, ICG_R_15, indocyanine green retention test after 15 min, CT, computed tomography, PTBD, percutaneous transhepatic biliary drainage, LTB, level of total bilirubin in the bile, TACE, trans-catheter arterial chemoembolization, ^99m^Tc-GSA, ^99m^T-galactosyl human serum albumin, ERCP, endoscopic retrograde cholangiopancreatography, ENBD, endoscopic nasobiliary drainage, ALPPS, associating liver partition and portal vein ligation for staged hepatectomy, Tumor thrombus in the bile duct, Biliary drainage, Hemobilia

## Abstract

**Introduction:**

Biliary drainage for patients with icteric-type hepatocellular carcinoma (HCC) is sometimes difficult, because the drainage tube makes contact with the tumor thrombus (TT) and effective drainage cannot be achieved due to hemobilia.

**Presentation of case:**

We performed endoscopic naso-biliary drainage for an icteric-type HCC patient; however, the serum bilirubin level was not decreased due to bleeding from the TT. Therefore, we performed tumor thrombectomy in the bile duct and transection of the right hepatic bile duct prior to right hepatectomy. After the first operation, the bilirubin level was decreased, and liver function was recovered so that the patient could undergo right hepatectomy 4 months after the first operation. The postoperative course was uneventful after the second operation and the patient was discharged from the hospital on the 30th postoperative day. The patient is well without recurrence 10 years after surgery.

**Conclusion:**

Biliary drainage is one of the key points for successful treatment of icteric-type HCC patients. A staged approach with initial tumor thrombectomy followed by hepatectomy should be considered as one of the options for icteric-type HCC.

## Highlights

• Obstructive jaundice due to tumor thrombus in the common bile duct with HCC is classified as icteric-type HCC.
• Biliary drainage for patients with icteric-type HCC is sometimes difficult.
• A staged approach with initial tumor thrombectomy followed by hepatectomy should be considered as one of the options for icteric-type HCC.

## 1 Introduction

Obstructive jaundice due to tumor thrombus (TT) in the common bile duct with hepatocellular carcinoma (HCC) is classified as icteric-type HCC [1]. Icteric-type HCC is considered a fatal condition; however, if curative surgery is achieved, long-term survival can be expected [[1], [2], [3], [4], [5], [6], [7]]. Biliary drainage is mandatory in icteric-type HCC patients who undergo major hepatectomy for the precise evaluation of liver function and prevention of cholangitis. However, biliary drainage is sometimes difficult in patients with icteric-type HCC with hemobilia. If hemobilia is present, there is no standard and effective treatment for icteric-type HCC patients. We report a case of icteric-type HCC successfully treated with a staged operation. This work has been reported in line with the SCARE criteria [8].

## 2 Presentation of case

**Fig. 1:**
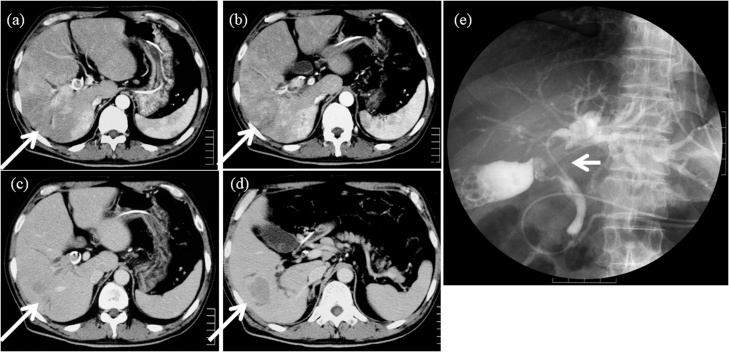
Computed tomography showed a tumor, 40 × 38 mm in diameter, enhanced in the arterial phase (a, b) and washed out in the portal phase (c, d) in segment 6 of the liver (arrow). The right intrahepatic bile duct was dilated and tumor thrombus in the bile duct was detected (arrow head c). Endoscopic retrograde cholangiography showed a filling defect from the right hepatic duct to the common hepatic duct (arrow, e).

**Fig. 2:**
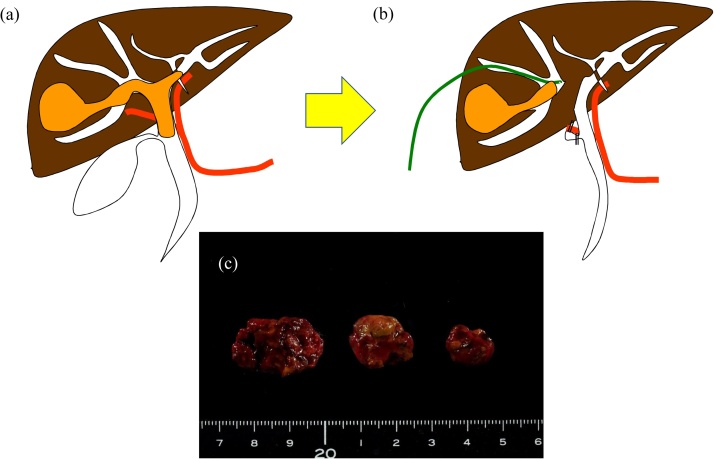
As initial operation, we performed thrombectomy in the bile duct, transection of the right hepatic bile duct, ligation of the right hepatic artery, and external drainage of the bile duct in the right lobe (a, b schema). Macroscopic findings showed tumor thrombus in the bile duct (c).

**Fig. 3:**
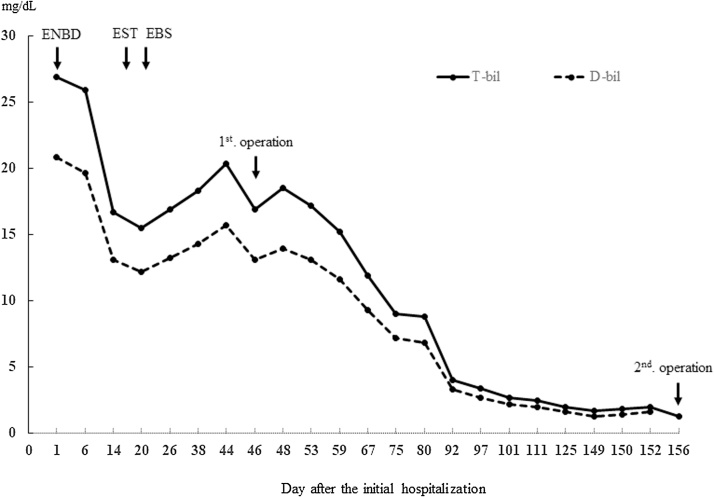
Trends in liver function after initial surgery.

**Fig. 4:**
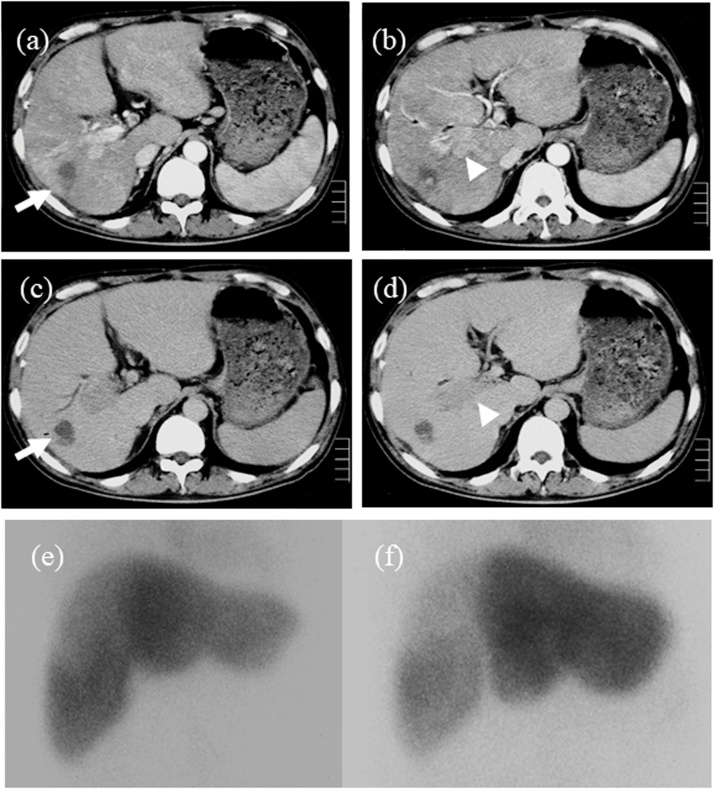
Computed tomography before the second operation showed the tumor in segment 6 decreased in size (arrow a, b: arterial phase, c, d: portal phase). However, a tumor thrombus had developed in the right main portal vein (arrow head b, d). ^99m^Tc-GSA scintigraphy revealed a reduced accumulation of GSA in the right liver (e, f).

**Fig. 5:**
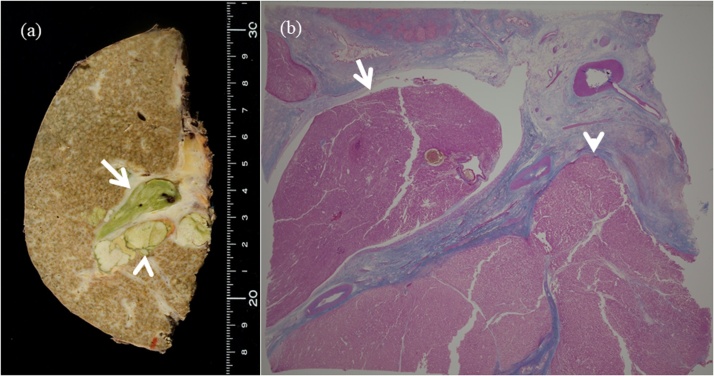
Macroscopic (a) and microscopic (b) findings showed tumor thrombi in the right hepatic bile duct (arrow head) and the right hepatic portal vein (arrow).

A 57-year-old man presented with jaundice. There was no past history of hepatitis or blood transfusion. Blood tests showed high levels of bilirubin, protein induced by vitamin K absence or antagonist-II (PIVKA-II) and carbohydrate antigen 19-9 (CA19-9). The indocyanine green retention test (ICG_R_15) could not be evaluated due to obstructive jaundice (Table 1a). Computed tomography (CT) scans showed a tumor 40 mm in diameter located in segment 6 with TT in the common hepatic bile duct (Fig. 1). Endoscopic retrograde cholangiography (ERCP) showed TT in the right hepatic duct and the common bile duct (Fig. 1). An endoscopic nasobiliary drainage (ENBD) tube was inserted in the left liver. ENBD was performed instead of percutaneous transhepatic biliary drainage (PTBD) due to the risk of tumor dissemination and bleeding, and insufficient dilation of the left intrahepatic bile duct. The level of total bilirubin in the bile (LTB) from the left liver as a future remnant liver was calculated at 1 week and 2 weeks after ENBD. LTB was calculated by multiplying bile density and volume obtained by ENBD [9]. One week after ENBD, the total bilirubin density was 16.9 mg/dL and the bile volume was 205 ml. Two weeks after ENBD, the total bilirubin density was 46.0 mg/dL and the bile volume was 235 ml. The LTB from the left liver increased from 34.6 mg to 108.1 mg. However, bleeding from the TT and cholangitis occurred so thatneither the patient’s general condition nor the serum level of bilirubin improved. One month after admission, the patient underwent thrombectomy in the bile duct, transection of the right hepatic bile duct and ligation of the right hepatic artery due to bleeding from the thrombus (Fig. 2). The bilirubin level immediately improved, and he was discharged from the hospital (Fig. 3). However, TT in the right portal vein developed (Fig. 4) and he was admitted to the hospital for a second operation. The serum level of total bilirubin was 1.3 mg/dl and ICG_R_15 was 34% at the second admission. Although the volume of bile could not be measured, the total bilirubin density in the bile from the left liver by ERCP was 40 mg/dL (Table 1b). ^99m^T-galactosyl human serum albumin (^99m^Tc-GSA) scintigraphy revealed a reduced accumulation of GSA in the right liver. In addition, left liver hypertrophy was observed on GSA scintigraphy (Fig. 4). We judged that right hepatectomy was possible because of the following reasons: (1) decrease in the serum level of total bilirubin, (2) hypertrophy of the left liver similar to portal vein embolization, (3) sufficient future remnant liver function on the basis of the total bilirubin density in the bile from the left liver. The patient underwent right hepatectomy for HCC. The total operation time was 326 min, and the total blood loss was 6264 g. The pathological findings yielded a diagnosis of moderately differentiated HCC with macroscopic portal vein and bile duct TT (Fig. 5). There was no intrahepatic metastasis, and the surgical margin was negative. The patient’s postoperative course was uneventful, and he was discharged on postoperative day 30 and he is well without recurrence 10 years after surgery.

**Table 1:** Laboratory data and liver function (a). Sequential data of the bilirubin level from the left liver (future remnant liver) (b).

(a)					
	1st. admission	Before hepatectomy		1st. admission	Before hepatectomy
Alb (g/dL)	2.8	2.2	WBC (/μL)	15290	10690
T-bil (mg/dL)	31.9	1.7	Hb (g/dL)	13.6	10.7
D-bil (IU/L)	22.5	1.3	Hct (%)	38.2	33.2
AST (IU/L)	67	64	Plt (/μL)	48.8 × 10^4^	21.6 × 10^4^
ALT (IU/L)	51	41			
LDH (IU/L)	425	204	PT (%)	66.7	62.4
ChE (IU/L)	125	NA			
ALP (IU/L)	784	701	HCV-Ab	negative	
γGTP (IU/L)	223	323	HBs-Ag	negative	
BUN (mg/dL)	21.3	14.4	HBs-Ab	negative	
Cr (mg/dL)	1.08	0.83			
CRP (mg/dL)	8.63	4.86	AFP (ng/dL)	42	103
			PIVKA-II (mAU/mL)	11736	NA
Child-Pugh	B	B	CEA (ng/mL)	1.6	NA
Liver damage	C	B	CA19-9 (U/mL)	2828	188
ICGR15 (%)	NA	34			

(b)			
	1 week after ENBD	2 week after ENBD	Before hepatectomy
ENBD bile volume (ml/day)	205	235	NA
Total bilirubin density from the left liver (mg/dL)	16.9	46.0	40.0
LTB from the left liver (mg)	34.6	108.1	NA

LTB: The level of total bilirubin in the bile.

## 3 Discussion

TT in the common hepatic bile duct causes obstructive jaundice, and is referred to as icteric-type HCC [1]. According to a recent report, 46.7% of HCC patients with TT in the right or left hepatic duct or the common hepatic bile duct presented with jaundice. Biliary drainage was performed in 83.3% of patients with jaundice and concurrent hemobilia was observed in 26 of 120 (22%) patients with jaundice [6].

Staged hepatectomy is usually performed for patients with unresectable bilobular liver metastasis from colorectal cancer. Portal vein ligation and associating liver partition and portal vein ligation for staged hepatectomy (ALPPS) are performed to increase remnant liver volume. The purposes of the initial operation were the control of hemobilia and effective biliary drainage; therefore, thrombectomy in the bile duct, transection of the right hepatic bile duct and ligation of the right hepatic artery were performed. Trans-catheter arterial chemoembolization (TACE) was one of the options instead of ligation of the right hepatic artery; however, Xiangji et al. reported that radical hepatectomy and removal of TT in the bile duct had a better prognosis than TACE alone in 184 patients with icteric-type HCC [10].

There are few reports on staged hepatectomy after thrombectomy in the common hepatic duct. Tsuzuki et al. commented on a staged operation for HCC with jaundice. They performed intrahepatic biliary drainage under local and general anesthesia, and emphasize that biliary drainage and controlling cholangitis were the keys to successful major hepatectomy [11]. Thrombectomy under choledochotomy and biliary drainage is considered one of the options for patients with icteric-type HCC.

Our patient had TT with hemobilia so that effective drainage was difficult. We could not perform right hepatectomy at the first operation due to high bilirubinemia and cholangitis. The patient’s condition improved immediately after the first operation and right hepatectomy could be performed successfully during the second surgery. If major hepatectomy is intended, estimation of the future remnant liver function is very important to avoid postoperative liver failure. However, for icteric-type HCC with high bilirubinemia, accurate estimation is difficult. In this case, ICG_R_15 was 34% and Child-Pugh was B after initial surgery with decreased serum level of total bilirubin. ^99m^Tc-GSA scintigraphy revealed a reduced accumulation of GSA in the right liver compared with preoperative images. In addition, left liver hypertrophy was observed on GSA scintigraphy (Fig. 4). CT showed a rapid increase in the TT in the portal vein from the right branch to the main trunk (Fig. 4), resulting in a situation similar to portal vein embolization. Furthermore, ERCP was preformed and bile juice from the future remnant liver was collected before the first and second surgery. Higuchi et al. reported that the preoperative bile level of total bilirubin in the predicted remnant liver can be used as a predictor for safe hepatectomy with obstructive jaundice [9]. In this case, the volume of bile could not be measured, but the total bilirubin density in the bile from the left liver by ERCP was 40 mg/dL before the second surgery (Table 1b). In addition to these, other liver functions were relatively well maintained (Table 1a).

Bile duct resection for bile duct thrombus of HCC is controversial. A recent article showed that surgical outcomes were better in patients who underwent bile duct resection [6]. However, Yamamoto et al. recommended preserving the bile duct for treatment of recurrences in the remnant liver [12]. Our patient, whose bile duct was preserved, is well 10 years after right hepatectomy. More discussion is required on whether to preserve or resect the bile duct for bile duct thrombus.

## 4 Conclusions

Biliary drainage is one of the key points for successful treatment of icteric-type HCC patients. A staged approach with initial tumor thrombectomy followed by hepatectomy should be considered as one of the options for icteric-type HCC.

## Funding

This study had no funding.

## Ethical approval

This study was not applicable for ethical approval.

## Consent

Informed consent was obtained from the patient for the publication of this case report.

## Authors’ contributions

All authors were involved in the preparation of this manuscript. TO, TY are the surgeons who operated on the patient. The manuscript was drafted by TY, YT, MY and MY supervised the preparation of this case report. All authors have read and approved the final manuscript.

## Registration of research studies

This study was registered in our institute.

## Guarantor

All authors are guarantors for this study.

## Provenance and peer review

Not commissioned, externally peer-reviewed.

## Declaration of Competing Interest

All authors have no conflicts of interest.
